# Interactions of unconjugated bilirubin with vesicles, cyclodextrins and micelles: New modeling and the role of high pKa values

**DOI:** 10.1186/1471-2091-11-16

**Published:** 2010-03-29

**Authors:** Pasupati Mukerjee, J Donald Ostrow

**Affiliations:** 1 School of Pharmacy, University of Wisconsin, 777 Highland Ave., Madison, WI, 53705-2222 USA; 2 GI/Hepatology Division, Dept. Medicine, Box 356424, Univ. Washington School of Medicine, 1959 NE Pacific St., Seattle, WA 98195-6424, USA

## Abstract

**Background:**

Unconjugated bilirubin (UCB) is an unstable substance with very low aqueous solubility. Its aqueous pKa values affect many of its interactions, particularly their pH-dependence. A companion paper shows that only our prior solvent partition studies, leading to pKa values of 8.12 and 8.44, met all essential requirements for valid pKa determinations. Other published values, generally lower, some below 5.0, were shown to be invalid. The present work was designed to derive suitable models for interpreting published data on the pH-dependent binding of UCB with four agents, mentioned below, chosen because they are not, themselves, sensitive to changes in the pH range 4-10, and the data, mainly spectrometric, were of reasonable quality.

**Results:**

These analyses indicated that the high pKa values, dianion dimerization constant and solubilities of UCB at various pH values, derived from our partition studies, along with literature-derived pH- and time-dependent supersaturation effects, were essential for constructing useful models that showed good qualitative, and sometimes quantitative, fits with the data. In contrast, published pKa values below 5.0 were highly incompatible with the data for all systems considered. The primary species of bound UCB in our models were: undissociated diacid for phosphatidylcholine, dianion for dodecyl maltoside micelles and cyclodextrins, and both monoanions and dianion for sodium taurocholate. The resulting binding versus pH profiles differed strikingly from each other.

**Conclusions:**

The insights derived from these analyses should be helpful to explore and interpret UCB binding to more complex, pH-sensitive, physiological moieties, such as proteins or membranes, in order to understand its functions.

## Background

The true pKa values of unconjugated bilirubin (UCB) are an important determinant of the proportion of the three ionization species of UCB in present in solution, and of the overall aqueous solubility of UCB, at any given pH value [1]. In a companion paper [2] we re-examined many published studies that assessed pKa values for UCB in simple solutions, determined by a wide variety of methods. We critically assessed the reliability of the methods used, in relationship to minimal criteria for validity, as well as other considerations. We summarized the deficiencies in the many reports which suggested that pKa values of UCB were below 7.0 and even below 5.0 (see Table eight in Boiadjiev *et al*. [3]). The only experiments which fulfilled all the validity criteria were our solvent partition data [4,5], which indicated that the two pKa values were much higher, 8.12 and 8.44.

pKa values of UCB are clearly of great importance in determining the effects of pH on the interactions of UCB with other molecules [1]. In the present paper, we critically re-examine and reinterpret several reports dealing with the effects of varied pH on the interactions of UCB with phospholipid vesicles, cyclodextrins, and dodecylmaltoside and bile salt micelles. Some general approaches have been developed to deal with the binding equilibria involved. We show that these studies are incompatible with proposed low aqueous pKa values for UCB. In several cases, the reinterpretations, using high pKa's and incorporating the effects of binding ratios, self-association and pH-dependent supersaturation, lead to some interesting new models and findings.

## Methods

### Criteria for Acceptability and Pitfalls in Interpretation

Studies of UCB binding vs. pH should meet the same criteria of validity proposed in the companion paper that deals with UCB alone in simple systems [2]: 1) The UCB is pure; 2) There should be no significant degradation of UCB; 3) Measurements are made after a rapidly achieved equilibrium; 4) Unbound UCB concentrations are below to minimally above aqueous saturation; 5) The pH range studied should encompass all suggested pKa values of UCB, and sufficient data points should be available to permit mathematical modeling.

In addition, for studies of the effects of pH on UCB binding: 6) The large molecule or components of the aggregate must also be purified; 7) Changes with pH in the ionization and/or conformation of constituent regions of the large molecule or aggregate must not affect its intrinsic affinity for each UCB species. This last criterion renders it difficult to perform detailed mathematical modeling of pH effects on interactions of UCB with proteins, natural membranes and other biologically-relevant systems, which are often impure and include molecules whose conformation and binding properties for UCB are affected by pH in incompletely known ways.

Further discussion is needed regarding criterion 4, above, including the thermodynamic solubility of UCB crystals [4], and the possibility of stable supersaturation [6,7]. Brodersen & Theilgaard [7], showed that, after extensive sedimentation at 100,000 *xg*, the UCB concentrations were 0.1 *μ*M at pH 7.40, 0.5 *μ*M at pH 7.83, 17 *μ*M at pH 8.05, and 34 *μ*M at pH 8.2. Solubility calculated from our partition data [4] was 0.062 *μ*M at pH 7.40, in reasonable agreement with the data of Brodersen's & Theilgaard [7], but our parition-derived solubilities at the higher pH values were considerably lower than theirs: 0.084 *μ*M at pH pH 7.83, 0.112 *μ*M at pH 8.05, and 0.148 *μ*M at pH 8.2. Thus, for reasons discussed previously [4,6,7], stable supersaturation with UCB, up to fairly high UCB concentrations, may be expected at pH values of 8.0 and above, particularly with the short time intervals between solution preparation and data gathering in most spectroscopic studies. At modestly high concentrations of UCB, such supersaturation may actually be promoted by amphipathic additives, for example bile salts [8,9]. In the reinterpretations attempted below, we have made comparisons of results expected in the presence and absence of supersaturation and assuming high vs. low pKa values for UCB.

### Selection of Publications for Further Analysis

To find papers for possible review, we electronically searched PubMed (1967-date), and ISI and Chemical Abstracts databases back to 1950, using the keywords "bilirubin, bile pigments, binding, hydrogen-ion concentration, pH, pKa, ionization", as well as the reference lists in papers thus discovered. To locate papers published earlier than 1968, we manually searched T.K. With's two comprehensive compendia of studies related to bilirubin [10,11]. After eliminating papers that dealt only with bile pigments other than biladienes, or with bilirubin ester conjugates, papers were then eliminated that failed to meet the majority of the criteria of validity summarized above. In line with validity criterion 7, above, we then focused on studies of interactions of UCB with host systems not expected to be sensitive to pH in the range of 5.5 to 10. These 12 papers and one abstract are summarized in Additional files 1, 2 and 3 (Tables S1, S2 & S3), which specify experimental deficiencies for each study. Finally, we confined our detailed analyses to the four best studies; binding of UCB to phosphatidylcholine (PC) [12], dodecylmaltoside micelles [13], β-cyclodextrins [14], and sodium taurocholate [15].

## Results and Discussion

### Ionization and Binding Equilibria of Unbound UCB Species

The equilibria governing the binding of unconjugated bilirubin (UCB) involve its three unbound species, the protonated diacid (H_2_B), the monoanions (HB^-^) and the dianion (B^=^), one or more of which binds independently to a larger host system [1], such as phospholipid vesicles, cyclodextrins, or micelles. For each UCB species, this binding can then be described in terms of a distribution ratio, K = mols bound/mols unbound (free).

**Figure 1:**
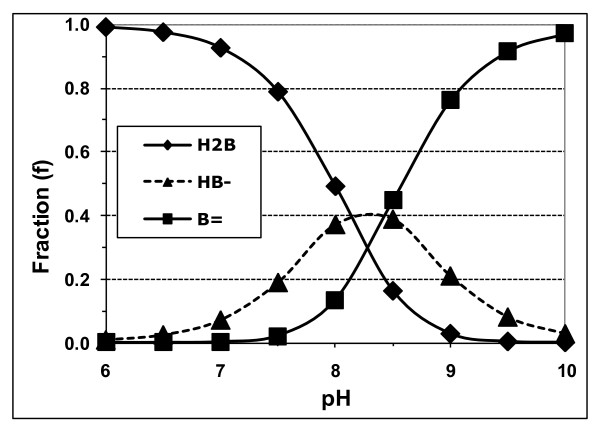
**Fractions (f) of unbound monomeric UCB at various pH values**. Calculated assuming high pKa values for UCB of 8.12 and 8.44 [4]. *Abbreviations: *H_2_B, UCB diacid; HB^-^, UCB monoanions; B^=^, UCB dianion. fH_2_B + fHB^- ^+ fB^= ^= 1.

**Figure 2:**
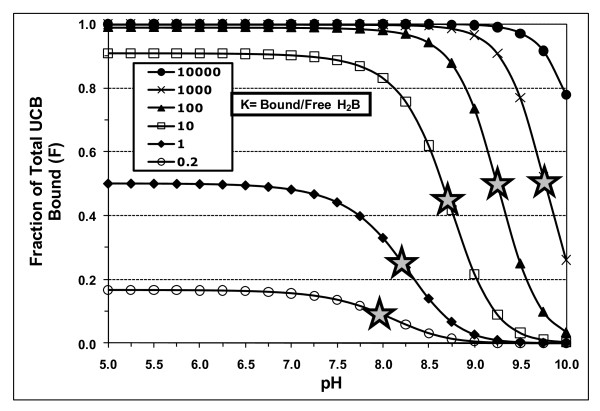
**Fraction of total UCB bound (F) at various pH values, assuming preferential binding of H_2_B: Effect of distribution ratio (K = bound/free UCB)**. Calculated assuming high pKa values for UCB of 8.12 and 8.44 [4]. H_2_B, fully protonated UCB diacid; shaded star, midpoint pH of titration curve.

**Figure 3:**
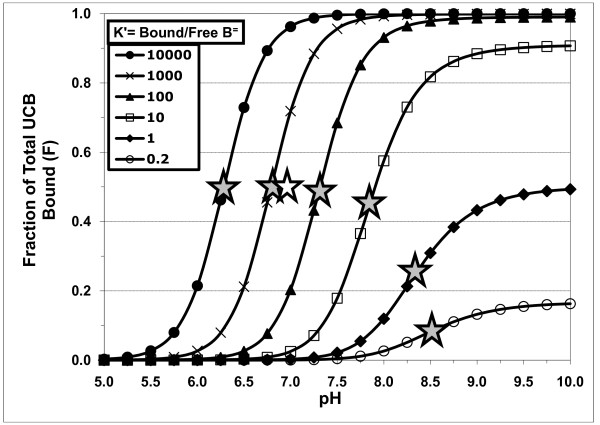
**Fraction of total UCB bound (F) at various pH values, assuming preferential binding of B^=^: effect of distribution ratio (K' = bound/free UCB)**. Calculated assuming high pKa values for UCB of 8.12 and 8.44 [4]. B^=^, UCB dianion; shaded star, midpoint pH of titration curve; open star, midpoint for TC binding (K' for B^= ^= 730, [21]).

**Figure 4:**
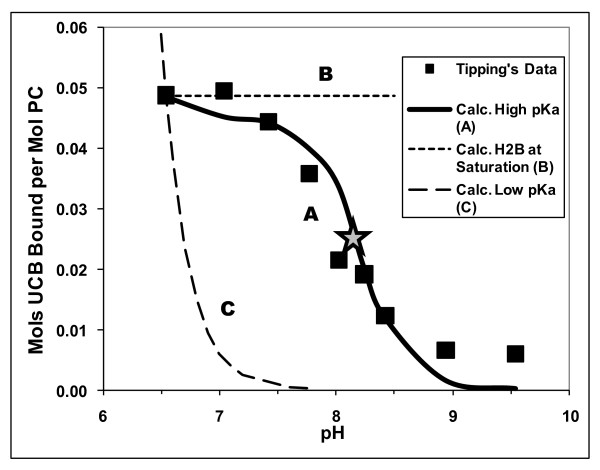
**Modeling UCB binding to phosphatidylcholine vesicles at various pH values**. Experimental data derived from Tipping *et al*., 1979 [12]. Curve A, calculated assuming pKa values for UCB of 8.12 and 8.44 and the dimerization constant for the B^=^ dianion of 0.26 *μ*M^-1 ^[4]. Curve B, calculated assuming constant maximum solubility of H_2_B of 51 nM [4]. Curve C, calculated assuming pKa values of UCB of 4.2 and 4.9 [3,17-20]. Shaded star, mid-point pH of binding curve A.

**Figure 5:**
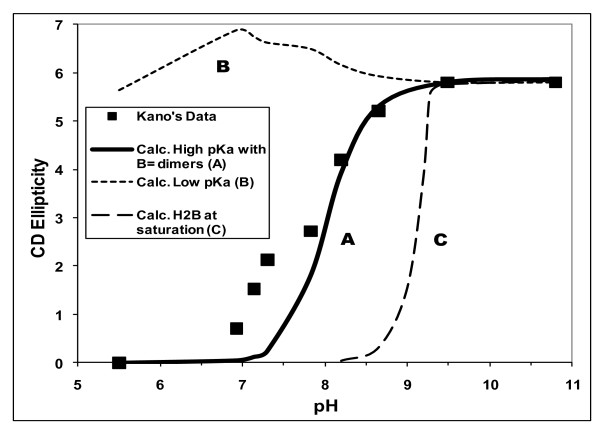
**UCB binding to β-cyclodextrin at various pH values**. Experimental data (black squares) from Kano *et al*., 1995 [14]. Curve A, calculated ellipticity assuming pKa values for UCB of 8.12 and 8.44, with B^= ^dimers [4]. Curve B, calculated ellipticity assuming pKa values of UCB of 4.2 and 4.9 [3,17-20]. Curve C, calculated ellipticity assuming constant maximum solubility of H_2_B of 51 nM [4].

**Figure 6:**
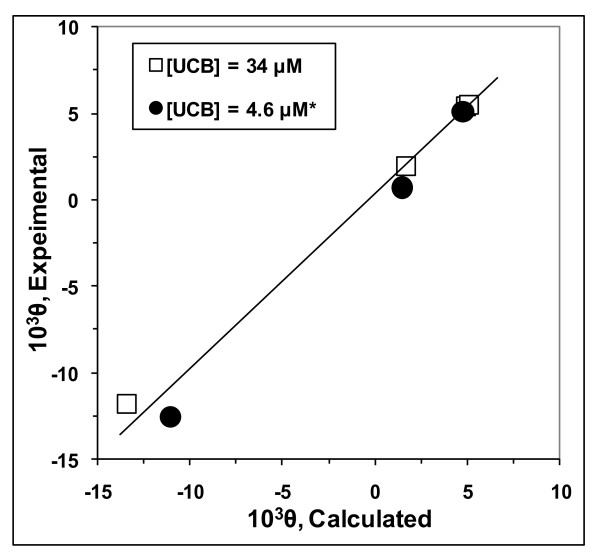
**Experimental vs. calculated CD ellipticities (10^3^θ) of UCB in 50 mM sodium taurocholate solutions. **Data for plotting taken from Table 2. *10^3^θ values, obtained with 4.6 *μ*M UCB, have been multiplied by the factor, 34/4.6 to render them comparable with data obtained at 34 *μ*M UCB. Experimental values are from D'Alagni M, *et al*. [15]. Calculated values are derived as described in the text and summarized in Table 2.

The ionization equilibria of the **unbound **species apply whether or not a binder is present. Thus, with a binder present, at any pH, the relative fraction (f) of each unbound **monomeric **species of UCB (fH_2_B, fHB^-^, and fB^=^) will be equal to f for that species, at the same pH, in an aqueous phase containing only monomeric UCB species [1]. Therefore, as described in the appendix in Hahm *et al*. [4], these unbound fractions are determined solely by the pH and the assumed pKa values of the monomeric species. The total concentration of unbound, monomeric UCB species (Bm) is given by Bm = [H_2_B] + [HB^-^] + [B^=^] = [H_2_B] × (1 + K_1_/[H^+^] + K_1_.K_2_/[H^+^]^2^). Note that these illustrative calculations and simulations (Figures 1, 2 and 3 and Table 1) deal only with pH effects on monomeric UCB species, so that fH_2_B + fHB^- ^+ fB^= ^= 1.00. Additional effects of the formation of B^= ^dimers [4] and supersaturation [7,9,16] have been considered below in the actual fitting of experimental data on UCB binding to phospholipid vesicles, cyclodextrins and micelles (Figures 4, 5 and 6 and Table 2).

**Table 1:** Fractions (f) of unbound UCB species at different pH values, assuming low pKa values of 4.2 and 4.9*.

pH	f_H2B_†	f_HB_^-^†	f_B_^=^†
**4.0**	0.5847	0.3689	0.0464
**4.5**	0.2639	0.5265	0.2096
**5.0**	0.0656	0.4137	0.5208
**5.5**	9.96E-03	0.1988	0.7913
**6.0**	1.17E-03	0.0735	0.9253
**6.5**	1.23E-04	0.0245	0.9753
**7.0**	1.25E-05	7.88E-03	0.9921
**7.5**	1.26E-06	2.51E-03	0.9975
**8.0**	1.26E-07	7.94E-04	0.9992
**8.5**	1.26E-08	2.51E-04	0.9997
**9.0**	1.26E-09	7.94E-05	0.9999
**9.5**	1.26E-10	2.51E-05	0.99997
**10.0**	1.26E-10	7.94E-06	0.99999

* Calculated assuming pKa values of UCB of 4.2 and 4.9 [3,17-20].

† Bilirubin species: H_2_B, diacid; HB^-^, monoanions; B^=^, dianion.

**Table 2:** Circular dichroism (ellipticity, 10^3^θ) of UCB in 50 mM sodium taurocholate solutions*

	[UCB]	**Exptl**.			Calculated
pH	*μ*M	10^3^θ†	f^s^HB^-^‡	f^s^B^=^ ‡	10^3^θ†
**7.23**	34.0	-11.80	0.1661	0.5790	-13.42
**7.35**	4.6	-12.60	0.1474	0.6780	-11.06
**8.13**	4.6	0.69	0.0345	0.9550	1.50
**8.15**	34.0	1.90	0.0330	0.9570	1.66
**9.12**	4.6	5.10	0.0037	0.9946	4.75
**9.35**	34.0	5.38	0.00217	0.9962	4.91
**11.60**	34.0	5.50	0.000012	0.9986	5.13

* Data from D'Alagni M, *et al*., 1994 [15]. For experimental problems, see Additional file 3, Table S3.

† 10^3^θ values obtained with 4.6 *μ*M UCB have been multiplied by a factor, 34/4.6, to render them comparable with 10^3^θ values obtained at 34 *μ*M UCB.

‡ f^s^HB^- ^and f^s^B^=^, fractions of total UCB bound to TC as monoanion and dianion respectively, calculated utilizing the partition ratios of each UCB species in water [4] and 50 mM TC [21], plus fitted 10^3^θ values for bound HB^- ^and B=, derived from modeling the experimental changes in 10^3^θ with pH, as described in the text.

Figure 1 shows the fractions of UCB species over the pH range of 4 to 10, using the high pKa values of 8.12 and 8.44 [4]. Table 1 shows the fractions derived by applying the low pKa values of 4.2 and 4.9, advocated in recent papers [3,17-20]. For such low pKa values, B^= ^is the dominant fraction at pH values above 7.0 (fB^= ^≥ 0.992, close to unity), with fH_2_B < 0.001 at pH values above 6.0 and fHB^- ^< 0.001 at pH values above 8.0. In stark contrast, for pKa values above 8.0 [4], the f values of H_2_B, HB^- ^and B^= ^are moderately high over wide ranges of pH and are of comparable magnitude at most pH values between 6 and 10 (Figure 1). At pH ≤ 6.0, only fH_2_B is above 0.99 and, over the pH range of 6.5 to 10.0, significant roles can be played by any of the three UCB species, each of which will show significant dependence on pH (Figure 1). The inescapable qualitative conclusion from these considerations is that numerous studies which show UCB interactions increasing with pH in range of 6.5-10.0 are far more compatible with the high pKa values of UCB (> 8.0) than the low ones (< 5.0).

### General Effects of Binding Affinities and pKa Values on Binding Curves

The values of pKa affect some characteristic features of the uptake and binding curves of UCB as a function of pH. As the pH increases, the equilibrium among the **unbound **species of UCB will shift to the more ionized forms (Figure 1, Table 1). The binding of UCB will, therefore, increase with pH if the preferentially bound species is B^= ^(as proposed for sodium taurocholate micelles [1,21]), but will decrease with pH if H_2_B is preferentially bound (as proposed for PC vesicles [4,12]).

The mid-point pH values of plots of binding vs. pH may shift significantly from the pKa of the unbound UCB ligand according to its affinity for the large molecule or aggregate, especially if the binding affinity for one of the three UCB species, H_2_B, HB^- ^and B^=^, is strongly dominant. This effect is easily modeled by assuming that only one of the three species is bound and that the unbound UCB concentrations are low enough for self-association of the unbound UCB species to be negligible. The proportions of the unbound species, H_2_B, HB^- ^and B^=^, are determined by the pKa values and the pH and calculated from the relevant terms in the model equation [4], as described above. If the relevant UCB species is not the only one bound, but is the predominant bound species, these analyses still apply qualitatively and the plots will closely resemble those derived by assuming that only a single UCB species binds.

Figures 2 and 3 show some simple representations of such modeling, using the high pKa values of 8.12 and 8.44 [4]; the fraction F = [UCB]_bound_/[UCB]_total _is plotted against pH. For Figure 2, it is assumed that only H_2_B binds, e.g. to a phospholipid vesicle [12]. The fraction F is calculated for values of the partition ratio K = [H_2_B]_bound_/[H_2_B]_free_, increasing from 0.2 to 10^4^. As expected (Figure 2), with increasing pH, F decreases sigmoidally due to the decreasing fraction (fH_2_B) of the unbound UCB diacid (Figure 1). For each curve, its mid-point pH, where the binding of H_2_B is 50% of the maximum, is represented in Figure 2 by a shaded star. These mid-point pH values increase from 8.255 for K = 1, to 8.780 for K = 10, 9.251 for K = 10^2 ^and 9.778 for K = 10^3^; for very low K values <<1 (e.g. for K = 0.2), the limiting mid-point pH is 7.994. This increase in the mid-point pH of the binding curves of H_2_B with increasing K is a general result that applies to binding to any large molecule and to any pKa values that are chosen. An example is binding of UCB to PC (Figure 4, curve A)

When the UCB dianion, B^=^, is the only interacting species, the uptake or binding is expected to increase with pH (Figure 3), due to the increasing fraction (fB^=^) of the unbound UCB dianion (Figure 1). Figure 3 plots F vs. pH at values of K' (= [B^=^]_bound_/[B^=^]_free_) of 0.2, 1, 10, 10^2^, 10^3 ^and 10^4^, assuming the pKa values are 8.12 and 8.44. The mid-point pH values of these sigmoidal curves (shaded stars), where binding of B^= ^is 50% of the maximum, decrease from 8.348 for K' = 1, to 7.863 for K' = 10, 7.315 for K' = 10^2^, 6.794 for K' = 10^3 ^and 6.287 for K' = 10^4^. The limiting value of the mid-point pH, when K' values are <<1 (e.g. K = 0.2), is 8.574. This trend of decreasing mid-point pH values with increasing K' is also a general result for binding of B^= ^to any large molecule for any pKa values chosen.

Many curves of UCB interactions as a function of pH show binding increasing with increasing pH (Figure 5; Additional files 1, 2 and 3, Tables S1, S2 & S3), indicating a primary role for the binding of B^= ^and dictating that the mid-point pH values must be **lower **than the true pKa values of unbound UCB (Figure 3). Therefore, the large number of these titration curves with mid-point pH values between 6 and 8 are compatible with high pKa values of 8.12 and 8.44 [4], and quite incompatible with pKa values below 5.0. If pKa values were low, e.g. 4.2 and 4.9 [3,17-20], curves showing increasing binding of UCB with increasing pH and, therefore, binding of B^=^, would yield mid-point pH values lower than about 5.0.

The mid-point pH values of sigmoidal titration curves, like those in Figures 2 & 3, are sometimes misinterpreted as average pKa values. This is clearly wrong, since all of the curves in Figures 2 &3 assume the pKa values of unbound monomeric UCB to be 8.12 and 8.44.

### UCB Binding to Phospholipid Vesicles (Additional file 1, Table S1)

Unilamellar vesicles of phosphatidylcholine (PC) are bilayers with a hydrophobic core of the fatty acid polymethylene chains, and hydrophilic exterior and interior surfaces composed of phosphryl-choline sidechains whose terminal -N^+^(CH_3_)_3 _groups are on the surfaces [22]. In the pH range 4 to 10, PC's are zwitterions, with both the amine and phosphoric acid groups fully ionized [23,24]; thus, changes in UCB binding to PC may be attributed to altered proportions of the three ionization states of unbound UCB in this pH range. Depending upon the molar ratios and experimental conditions under which the UCB and PC are mixed, either H_2_B or B^=^ may be the UCB species that is preferentially bound [25].

pH effects on the interactions of UCB with PC vesicles have been reported in a number of studies [12,16,22,25-27]. Many of them involve time-dependent processes in non-equilibrium situations. Some used PC systems containing fluorescent probes, which are difficult to interpret due to uncertainties regarding the localization of the probes and the nature of their interactions with UCB.

The report by Tipping *et al*. [12], which assessed binding of UCB to egg PC vesicles from the effects of binding on the spectrum of UCB, most fulfills the above criteria of an acceptable study (Additional file 1, Table S1), although neither the UCB (10 μM) nor the PC (100 μM) were purified. We have, therefore, amplified our previous analysis [4] of this study.

The data points in Figure 4 (black squares) show values of ñ (moles of UCB bound per mole of total PC) vs. pH over the pH range of 6.54 to 9.54, obtained from Figure 10 in the paper of Tipping *et al*. [12]. ñ decreases from a maximum of 0.049 as pH increases, indicating (see Figure 2) that the binding of H_2_B is the predominant interaction. The concentration of bound UCB (Bb = ñ × 100 μM) is then used to calculate the concentration of unbound UCB (Bf = 10 μM - Bb). Bf ranges from 5.1 *μ*M at pH 6.54 to 9.5 *μ*M at pH 9.54.

At each pH, the fraction of unbound H_2_B (= [H_2_B]f/Bt) in the presence of 100 μM PC [12], is assumed to be equal to the fraction of H_2_B ([H_2_B]/Bw) in the aqueous phase in the absence of PC, calculated using Equation four in Hahm *et al*. [4]) and applying the high pKa values of 8.12 and 8.44 and the dimerization constant for the B^=^ dianion of 0.26 *μ*M^-1^.

Assuming that unbound UCB is in a solution state and that H_2_B is the **only **species that binds to the PC yields a simple model of distribution of H_2_B between the free and vesicle-bound state, ñ = K" [H_2_B]f. Applying this equation to the 9 data points of Tipping *et al*. [12], yields a mean K" = 0.00932 ± 0.00053 *μ*M^-1^) and curve A in Figure 4). The fit of curve A with the experimental ñ values (black squares) is quite good (r = 0.953), and suggests that our assumptions are valid and that the H_2_B partition model is reasonable. The deviation of the data points from Curve A at the highest two pH values possibly reflects weak binding of the B^=^ dianion [16,22,27], which constitutes over 80% of the UCB species above pH 9.0.

Tipping *et al*. [12] reported that "The spectral differences developed within the time required to mix the solutions and start recording and were the same whether multilamellar or unilamellar vesicles were used". This rapid analysis and establishment of equilibrium are necessary conditions for our detailed interpretation of the data. With the total concentrations of UCB = 10 μM and PC = 100 μM (molar ratio of UCB/PC, 0.1) used in the pH-variation study, the unbound UCB concentrations (Bf) at equilibrium were modestly above the aqueous solubility of UCB at all pH values ≤ 9.0 [4]. If supersaturation were absent, the concentration of H_2_B would be at its saturation value over the entire pH range, and ñ should maintain its value of 0.049 at pH 6.54 (the horizontal dashed line B, in Figure 4). The experimental data indicate, therefore, that stable supersaturation was indeed likely present in these short term experiments.

If, by contrast, the low pKa values of 4.2 and 4.9 [3,17-20], are used to fit the ñ value at pH 6.54, a much higher value of K", 94 *μ*M^-1^, is needed to yield the calculated [H_2_B]. Using this constant and [H_2_B], determined from Bf and the low pKa values, yields curve C in Figure 4. As pH increases from 6.5 to 7, ñ decreases steeply, and is below 0.01 at a pH of 6.9 or higher. The low pKa values are, thus, not compatible with ñ vs. pH data determined for the binding of UCB by neutral PC vesicles [12]. Since, in this study, K ≈ 1 [12], the mid-point pH value of 8.1 (shaded star, Figure 4, curve A) would be close to the mid-point value of the curve for K = 1 in Figure 2, and thus compatible with our proposed high pKa values in the absence of a UCB binder [4].

Comments are warranted on an often-cited study, by Eriksen, *et al*., of the binding of UCB to egg PC vesicles [16]. The concentrations were 12.8 *μ*M UCB and 130 *μ*M PC. Figure two in that paper plots the changes with pH of the quenching by bound UCB of the fluorescence of diphenyl-hexatriene incorporated into the lipid bilayer, measured 3 hours after adding the UCB in an aqueous alkaline solution (pH 11). The sigmoidal curve exhibits decreasing fluorescence (increasing quenching by bound UCB) as pH increases, with a mid-point near pH 7.8. This evidence for increased affinity of UCB binding to PC as pH increases would indicate a primary role of binding of the dianion, B^=^. Since the dianion fraction for low pKa values is already 0.992 at pH 7.0 (Table 1), any significant increase in binding at higher pH values is incompatible with low pH values (see above). The increasing affinity of UCB for PC with increasing pH is in stark contrast to Tipping's data [12] and to our model based on uptake of H_2_B (above). However, examination of data in Table one of the Eriksen paper, reveals that results obtained "immediately" after adding the UCB at pH 7.4 and 8.3 are just the reverse of those obtained three hours later (as in their Figure two).

Eriksen's Figure one [16] indicates complex, pH-dependent time effects. Using the percent decrease in fluorescence, P, as a rough measure of the extent of binding of UCB, the data show a high value of P = 79% at pH 7.43 "immediately" after addition of UCB. Within one minute, there is rapid reversal of binding and P is decreased to 28%, followed by a far less rapid decrease in P to 23% after 3 hours. Results at pH 6.79 are similar. In contrast, at the highest pH in their Figure one, 8.34, the "immediate" value of P = 57% remains about the same for three hours. At the intermediate pH of 7.90, the "immediate" value of P = 73% increases a little over the first minute, remains about the same for four more minutes, and then decreases gently over the next two hours or so to a plateau of P = 22%. These complex time effects yield very different sequences of P values decreasing with pH: 7.43>7.90>8.34 for "immediate" measurements; the irregular sequence 7.90>8.34>7.43 between 1 and 20 minutes; 8.43>7.90>7.43 at 60 minutes; and 8.34>7.90 ≈ 7.43 at 3 hours. Some discrepancies among data in Eriksen's Table one, Figure one and Figure two [16], suggest irreproducible time effects.

These observations can be rationalized roughly on the basis of the progressive formation of aggregates of UCB, after the initial rapid binding of UCB. Cestaro *et al*. [22] have made a similar proposal, and Eriksen *et al*. [16] themselves warn that, as the pH falls, supersaturation of the aqueous medium with UCB must be avoided to prevent progressive formation of H_2_B aggregates. Their gradient ultracentrifugation data of 3 hour incubations (Figures three-d&e of Eriksen *et al*.) [16] showed large aggregates of UCB, with or without phospholipids, at pH 7.0 and smaller aggregates at pH 8.2. The dependence on pH, of the rates of formation of colloids and precipitates from supersaturated solutions of UCB itself, is clearly relevant. As discussed earlier, after extensive sedimentation at 100,000 *xg*, the measured supernatant concentrations of UCB at pH values of 7.40, 7.83, 8.05 and 8.2 were 0.1 *μ*M, 0.5 *μ*M, 17 *μ*M and 34 *μ*M respectively [7]. These are much higher than the calculated solubilities of UCB at the same pH values [4], which were low (0.062 *μ*M, 0.084 *μ*M, 0.112 *μ*M and 0.148 *μ*M) and increased only a little as pH increased. This indicates that the egg PC-UCB systems of Eriksen *et al*. [16] were highly supersaturated when first prepared, and that aggregates of UCB formed to relieve the supersaturation. This occurred to a much greater extent and much more rapidly at pH 7.43 than at pH 8.34, with intermediate behavior at pH 7.90. Any micro-precipitation of UCB, which may contain small proportions of PC, will reduce Bf and its interactions with PC vesicles. Vazquez *et al*. [28] have also reported significant reversal of UCB interaction with phospholipid membranes with time.

In view of the above considerations, and evidence that the uptake of UCB by PC vesicles is almost instantaneous (t_1/2 _= 2 msec) [29], we conclude that the "immediate" effects on P after addition of UCB to PC are least susceptible to time-dependent effects arising from the thermodynamic drive to decrease supersaturation. Indeed, even the "immediate" values of P may still be subject to some aggregation effects, particularly at pH 7.43. The qualitative trend in the "immediate" decrease of P with increasing pH is consistent with the results of Tipping et al. [12] and with our model, based on the binding of H_2_B and high pKa values for UCB. The conclusions are opposite to those given by Eriksen *et al*. [16], which were based on assumptions of binding of the dianion, B^=^, and low pKa values.

Other systems studied by Eriksen *et al*. [16] (their Table one and Figure one-b) also show exceptionally large and rapid decreases in binding of UCB with time at pH 7.4, intermediate behavior at pH 7.90, and minor effects at pH 8.34. These systems all contained 12.8 *μ*M UCB, mixed with various types of phospholipids ± cholesterol, at varied concentrations: egg PC, 150 *μ*M + cholesterol 15 *μ*M; phosphatidlyethanolamine, 106 *μ*M; phosphatidlyethanolamine, 102 *μ*M + cholesterol 10 *μ*M; sphingomyelin, 32 *μ*M; and phosphatidylserine, 55 *μ*M. The qualitative interpretations above apply to all of them. The data points are limited, and some of these systems involve charge effects, which introduce further uncertainties. In all systems, after 3 hours, UCB binding is greater at pH 8.3 than at pH 7.4. The "immediate" results, however, show a stronger reverse effect of pH, P = 82% at pH 7.4 vs. 25% at pH 8.3, for the egg PC-cholesterol system as compared to the egg PC system. They also show lower binding to sphingomyelin at the higher pH, indicating predominant binding of H_2_B. This common pattern leads us to conclude that the complex pH-dependence of precipitation of UCB from supersaturated solutions can lead to highly anomalous pH-dependence of UCB interactions with phospholipids.

The study of UCB binding to gangliosides by Vazquez *et al*. [30], reported little change in UCB binding going to pH 7.4 to 8.0, but a small increase in going from pH 7.0 to 7.4, suggesting an important role for UCB anions. Assuming low pKa values, the unbound dianion fraction changes only from 0.992 at pH 7.0 to 0.999 at pH 8.0, whereas the monoanion fraction decreases by a factor of 10 (Table 1). Therefore, these observations are incompatible with low pKa values, whereas with high pKa values, both the mono- and dianon fractions increase significantly over this pH range (Figure 1). Other problems with this study (Additional file 1, Table S1) are that: the systems were highly supersaturated with UCB, the UCB was neither purified nor protected against degradation, equilibrium was not attained rapidly, and the pH range studied was insufficiently broad to derive pKa values.

Zucker and Gössling used stop-flow kinetics to examine the rates of uptake of BSA-bound UCB by cultured HepG2 cells [26]. They found an inverse correlation between pH and the rate of UCB flip-flop, which they showed to be due to pH effects on the rates of dissociation of UCB from albumin and from the membrane bilayer. They concluded that "the identification of an inflection point at pH 8.1 is indicative of a pKa value for bilirubin in this range". Although this seemingly supports our contention that the pKa's of UCB are above 8.0 [4], these are kinetic and not equilibrium data, involve BSA (which is pH-sensitive in this range), and measure diffusion across a complex hepatocyte membrane. Studies of binding of UCB to whole cells [31], or natural membranes (e.g. red cell ghosts, mitochondria, synaptosomes) [28,32-35], likewise cannot be used to evaluate unequivocally the role of pKa values for UCB. These heterogeneous structures contain a mix of phospholipids, as well as proteins and other components, which may ionize and change conformation in the pH range studied and have unknown effects on membrane structure.

### Interactions of UCB with Dodecyl Maltoside Micelles (Additional file 2, Table S2)

Non-ionic long-chain alkyl-saccharides, such as octylglucoside (C_8_G) or dodecylmaltoside (C_12_M), are readily purified and form micellar systems that are suitable for studies of the influence of pH on the uptake of UCB. Binding probably involves interactions of the -OH groups of the saccharide with the ionized -COO^- ^groups of UCB, and induces marked changes in the ellipticity of the bound UCB [13].

Kano *et al*. [13] showed that solubilization of UCB (20 *μ*M) in 1 mM C_12_M micelles (their Figure five) generated no circular dichroism effects from pH 4.5 to 6.2. (Note that their legend for Figure five misidentifies the saccharide as C_8_M, octylmaltoside; the text indicates it is C_12_M that was studied.) Kano's Figure three shows that UCB does not interact with C_8_G below the c.m.c. of 22 mM. The same would be expected of C_8_M, whose c.m.c. value should be similarly high, but the concentration of the alkyl-saccharide used in their Figure five is 1 mM, well below the presumed c.m.c. of C_8_M.) Above pH 6.2, the observed ellipiticity increased steeply with rising pH up to about pH 8.5, and leveled off to a constant value from pH 8.8 to 10. Since the conformation of the non-ionic C_12_M is unaffected by pH, their titration curve reflects increased binding of UCB dianion (B^=^) as the proportion of unbound dianion, fB^=^, increases with increasing pH.

The rise in ellipticity with pH has a mid-point of about pH 7.5. Reference to our Figure 3 shows that this is compatible with high pKa values of 8.12 and 8.44 and a UCB distribution ratio K (bound/free) between 10 and 100. If the low pKa values of 4.2 and 4.9 are invoked, the midpoint pH value would have to be lower than about 5, as discussed above. Moreover, at pH 6.5, the fraction of UCB dianion, fB^=^, would already be 97.5%, yet the observed ellipticity is near zero at that pH. In addition, the steep rise in ellipticity observed above pH 6.5 would not be expected. Thus, only the higher pKa values are compatible with the observed data.

### Interactions of UCB with Cyclodextrins (Additional file 2, Table S2)

Kano *et al*. [14] and Lightner *et al*. [36] studied CD of UCB in solutions of β-cyclodextrin (β-CDx) as a function of pH. Both studies used purified UCB and were conducted within minutes of mixing UCB with the cyclodextrins; Kano's studies also took precautions to minimize oxidation and photo-oxidation of the pigment. In each study, the molar circular dichroism absorption coefficient (Δε) was minimal at pH 6.0 and increased steeply as pH increased above pH 7.0, indicating that UCB dianions are the primary interacting species. Lightner's systems [36] were even more supersaturated than Kano's and measurements were provided only at integral pH values from 6 to 10, rendering Kano's data [14] preferable for analysis.

In the study by Kano *et al*. of UCB binding to β-CDx [14], no ellipticity was observed at pH 5.5, but Δε increased sigmoidally as pH increased from 5.5 to 9.5, with a steep slope between pH 7.0 and 8.5, and no further change between pH 9.5 and 10.8 (their Figure 3). Chiral recognition effects, registered at several pH values with the protonated 1-amino-β-cyclodextrin as the host molecule, (their Figure three [14]), support the conclusion that predominantly UCB dianion was bound.

At pH 10.8, an affinity constant of 23 L/mol was reported for UCB binding to β-CDx, indicating that 4.67 *μ*M, or 18.7% of the total [UCB] (25 *μ*M), was bound to 10 mM β-CDx at that pH. Assuming that the UCB binding is confined to B^=^, and that binding of UCB is proportional to the concentration of unbound, monomeric B^=^, the amount of UCB bound at lower pH values can be calculated using Δε values relative to Δε at pH 10.8. It is then possible to calculate Bf and, akin to modeling the data on binding of UCB to PC as described above, to apply Equation four of Hahm *et al*. [4] to calculate the unbound, monomeric dianion concentration, [B^=^], at each pH, assuming different pKa values.

Assuming the high pKa values of 8.12 and 8.44 and the dianion dimerization constant of 0.26 *μ*M^-1 ^[4] in calculating [B^=^]f from Bf, the four data points at pH values of 10.8, 9.5, 8.65 and 8.2 showed an excellent proportionality between Δε and [B^=^] using the equation Δε = Q × [B^=^]f, where the constant, Q = 1.099 ± 0.018 (n = 4, r = 0.974, S.D. = 0.17). Curve A in Figure 5 shows the results of fitting all the data to the above equation, Δε = 1.099 × [B^=^]. Curve A gives a reasonable fit for the experimental Δε at the five highest pH values (7.83 and above) and, of course, the very low pH of 5.5, where Δε = 0. Bf was below calculated solubilities at both pH 10.8 and 9.5, but there was likely stable supersaturation of the unbound UCB phase at the three lower pH values, based on previous observations [7], as discussed earlier. The rapid decrease in Δε below pH 8.65 is primarily due to the rapid decrease in the fraction of unbound monomeric UCB that exists as the dianion (fB^=^). The systematic discrepancies between the data and Curve A in the pH range of 7.3 to 6.9 (Figure 5) can be ascribed to many factors that are difficult to evaluate, including self-aggregation of the more highly supersaturated phase of unbound UCB (e.g. R = 369 at pH 7.0).

In comparison to the reasonable representation of the Δε data using pKa values above 8.0, the results obtained are very different (Figure 5, Curve B) if the lower pKa values of 4.2 and 4.9 [3,17-20], are used to calculate [B^=^] from Bf. The proportionality constant K, relating Δε to [B^=^] was determined by fitting the data at the pH values of 9.5 and 10.8, where the solutions are undersaturated with unbound UCB. K was then used to calculate Δε from the estimated [B^=^] at other pH values. Since the dianion fraction (fB^=^) of unbound UCB would be close to unity at pH 7.0 or above, and have the value of 0.79 even at pH 5.5, little variation is expected in [B^=^] and, therefore, in Δε over the alkaline pH range. This is shown by Curve B in Figure 5, which does not fit Kano's Δε vs. pH data [14]. This indicates that the low pKa values are highly incompatible with the experimental observations. The same conclusions apply to the data of Lightner *et al*. [36], which show similar pH dependence of binding at alkaline pH.

From the mean fit of the Δε data at pH 9.5 and 10.8 to the concentrations of [B^=^] calculated using pKa values of 8.12 and 8.44 and the dianion dimerization constant of 0.26 *μ*M^-1^, it is possible to calculate Δε from [B^=^] at lower pH values, where total solution concentrations are above solubility, by assuming that all solutions are at true saturation, with the excess [B^=^] forming a suspension. For such systems, [B^=^] = 0.051 × 10^(2pH-16.56)^. Here, 0.051 *μ*M is the solubility of H_2_B and 16.56 = pKa_1 _+ pKa_2 _[4,6]. The calculated curve C (Figure 5) shows a steep drop in Δε below pH 9.3. Curve C has little resemblance to the experimental data, suggesting that, at most pH values, a stable supersaturation was probably important in these relatively short-term experiments.

### Interactions of UCB with Bile Salts (Additional file 3, Table S3)

Taurine-amidates of bile salts, which have pKa values near 1.0, are the only bile salts which are fully ionized throughout the pH range from 4 to 10 [37], and are thus most suitable to assess the roles of pH and pKa values on UCB binding. Rapid solvent partition of purified UCB from undersaturated chloroform solutions into buffered aqueous solutions of 50 mM taurocholate (TC) (at least 80% in micelles), at ionic strength = 0.15, was performed over the pH range of 6.0 to 9.5 [1,21]. The data were fitted to the equation log P = log Po + [1 + 10^(pH-A) ^+ 10^(2pH-B)^], where Po is the partition ratio for H_2_B, and the values for A and B were 7.36 ± 0.5 and 14.08 ± 0.05 respectively.

Solubilities of UCB in 50 mM TC [21] and in water [4] were calculated from the fitted P data and an estimated solubility of UCB in chloroform of 1.14 mM [9]. (As argued before [4,6], a better estimate of the solubility of UCB in chloroform is 0.88 mM, which will decrease the estimated UCB solubilities in 50 mM TC and water by 23%, but not affect the shape of the titration curves.) Using the high aqueous pKa values of 8.12 and 8.44, the total and unbound concentrations of each UCB species in 50 mM TC was calculated from the solubilities in 50 mM TC/chloroform [21] and water/chloroform [21] respectively; their difference equaled the concentration of the bound species. From these, the distribution ratios (bound/free) of the three UCB species were calculated to be 1.41 for H_2_B, 12.9 for HB^-^, and 730 for B^=^, indicating predominant binding of B^= ^[21], as concluded previously by Kano *et al*. [38]. From the relative contributions of bound B^=^, HB^- ^and H_2_B, the apparent pKa values of UCB associated with the TC were estimated: 7.16 ± 0.5 for pKa_1_, 6.69 ± 0.5 for pKa_2 _and 13.85 ± 0.05 for the sum of pKa_1 _and pKa_2_, which is derived with somewhat greater precision.

The relationship of these pKa values, lower than the aqueous values of 8.12 and 8.44, to micelle-water distribution ratios for H_2_B, HB^- ^and B^=^, has been discussed in our companion paper [2]. pKa values in the bile salt systems in the range of 6 to 7 have also been reported from studies using micellar electrokinetic capillary chromatography [39,40]. Since these authors did not determine micelle-water distributions, these were average values for the whole system, but appear to be compatible with Hahm's pKa values in 50 mM TC derived by solvent partition [21]. Utilizing essentially the same equations [1,40] relating changes in pKa values resulting from uptake in micelles and micelle-water distribution ratios for H_2_B, HB^- ^and B^= ^mentioned above [21], the pKa values of 6 to 7 in 50 mM TC are thus compatible with the high pKa values in water [4].

CD studies have been performed with unpurified UCB in 50 mM solutions of various bile salts [15,41,42]. In many cases, the UCB concentrations used appear to be above saturation. For 50 mM sodium taurocholate (TC), calculated Bf was above aqueous phase saturation at pH below 7.5 when total UCB was 4.6 *μ*M and at pH below 8.3 when total UCB was 34 *μ*M. Bile salts, however, have been shown to promote extensive, stable supersaturation of UCB [8,9]. In systems of UCB in 50 mM TC [15], the observed ellipticities were reasonably proportional to the two UCB concentrations, even though the calculated solubility in 50 mM TC was exceeded at several pH values. Similar proportionalities are seen over higher ranges of concentration of sodium glycocholate [15] and sodium deoxycholate [41] at pH values of 7.0 and above.

Since background information is available for 50 mM TC [21] and TC is expected to show no dependence upon pH, we have attempted a semi-quantitative interpretation of the pH effects for the ellipticity study of 4.6 *μ*M and 34 *μ*M UCB in 50 mM TC [15]. We assumed stable supersaturation in these short-term experiments (30 min or less) at pH values of 7.23 and higher. Table 2 presents ellipiticity values at 458 nm, estimated from their published graphs. The three measurements at 4.6 *μ*M UCB have been multiplied by the factor, 34/4.6 to render them comparable to measurements at 34 *μ*M UCB. The ellipticities are large and negative at the two lowest pH values of 7.23 and 7.35, become positive at pH about 8.1, and then show higher positive values as pH increases to 9.1 and above. This general trend was exhibited by all the bile salts studied [15,41,42].

As noted earlier, equations relating partition coefficients to pH for 50 mM TC [21] and water [4] allow the calculation of the fractions of total UCB solubilized by the TC as H_2_B, HB^- ^and B^=^. The pH-dependence of the ellipticity values at 458 nm suggest strongly that the interactions with TC aggregates of the solubilized HB^- ^cause negative ellipticities whereas interactions of the solubilized B^= ^cause positive ellipticities. Table 2 shows the estimated fractions of total UCB (34 *μ*M) that are bound to TC as HB^- ^and B^=^. As expected, as pH increases, the fraction of total UCB that is bound to TC as HB^- ^(f^s^_HB_^-^) decreases and the fraction bound as B^= ^(f^s^_B_^=^) increases. In order to test our model, we use the equation below in which the constants A* and B* are measures of the effectiveness of (f^s^_HB_^-^) and (f^s^_B_^=^) in determining the value of 10^3^θ:

The experimental ellipticity values in Table 2 are well described by this equation: n = 7, r = 0.992, SD = 1.1, A* = -98.7 ± 5.6, B* = 5.24 ± 0.54. Figure 6 shows that, at total UCB concentrations of 4.6 and 34 *μ*M, the experimental ellipticities are in reasonable concordance with calculated ellipticities. Note that the two most positive ellipticity values for 4.6 *μ*M UCB, at pH 8.13 and 9.12, and for 34 μM UCB at 9.35 and 11.60 were measured in unsaturated solutions. The significant changes in ellipticity at these high pH values are clearly due to significant changes in (f^s^_HB_^-^) and (f^s^_B_^=^) over this alkaline pH range. Remarkably, the measured ellipticity increased significantly, by about 2%, between pH 9.35 and 11.60, which is matched by a calculated increase in ellipticity of about 4%. Since, if low pKa values are assumed (Table 1), f_B_^= ^is already close to unity at pH 8.5 (0.9997), and the changes in ellipticity at pH values above 9.0 are qualitatively incompatible with the low pKa values for UCB proposed by others [3,17-20]. The model proposed here for representing the circular dichroism values of UCB in 50 mM TC appears to be new and is likely to be useful also for other bile salt systems.

## Conclusions

The analyses and interpretations presented above show that the high pKa values of 8.12 and 8.44 [1,4-6] are far superior to low pKa values of 4.2 and 4.9 [3,17-20] in rationalizing experimental data for the effects of pH on interactions of UCB with PC vesicles, cyclodextrins, and micelles of dodecyl maltoside and bile salts. The approaches outlined also demonstrate and take into account the possibly important roles of self-association of B^= ^and pH-dependent supersaturation effects. They support further the conclusions of our companion paper [2] that analyzes the effects of pH on various properties of UCB alone in aqueous and organic solvent systems. That paper demonstrated that only our solvent partition studies [4,5] met all the requirements for valid experiments when using a poorly-soluble, unstable compound, such as UCB. Together, the two present papers clearly indicate that the pKa values of UCB are well above the pKa values of simple carboxylic acids (usually about 4.5). Theoretically these remarkably high values may result from the combined interactions of three factors that result from the unique, complex internal hydrogen-bonding of UCB, as presented elsewhere [5].

We do not claim that the high pKa values of 8.12 and 8.44, derived from our solvent partition study of UCB [4], are exact. The accuracy of these model-derived high pKa values of UCB is given on page 1131 of that paper. These values (mean ± S.D.) are: pK'_1 _= 8.12 ± 0.23; pK'_1 _+ pK'_2 _= 16.56 ± 0.10; pK'_2 _= 8.44 ± 0.33. That paper also notes that "the analysis yields a less accurate estimate of the individual pKa values than of their sum, pK_1 _+ pK_2_". The errors in these pKa values are insignificant when compared with the difference of 7.5 pH units between the sum (pK_1 _+ pK_2_) of these high pKa values, and the sum (9.1) of the low pKa values of 4.2 and 4.9 proposed by others [3,17-20], with which our high pKa values were compared.

It should thus now be clear that such high pKa values not only acceptably explain the experimental findings on the effects of pH on the properties and interactions of UCB, but that the often quoted low pKa values [3,17-20], are incompatible with the experimental data. The implications are not trivial, since they invalidate older, but still widely held, interpretations and modeling of the binding and cytotoxicity of UCB [43-47] that were based on the assumptions that pKa values were below 5.0 and that B^= ^was, therefore the dominant species of unbound UCB in the physiological pH range (Table 1). More recent reviews are available [1,48,49] that are based on the realistic pKa values above 8.0, and the consequent predominance of H_2_B among the unbound UCB species at physiological pH values (Figure 1).

## List of Abbreviations Used

UCB: unconjugated bilirubin; H_2_B: UCB diacid; HB^-^: UCB monoanions; B^=^: UCB dianion; fH_2_B: fHB^-^, and fB^=^, relative fractions of the unbound monomeric UCB species; F: [UCB]_bound_/[UCB]_total_; Bf: unbound (free) UCB concentration; R: the UCB saturation ratio = Bf/estimated solubility of UCB at a given pH; K: distribution ratio = mols bound/mols unbound (free); PC: phosphatidylcholine; c.m.c., critical micellar concentration; C_12_M: dodecylmaltoside; C_8_M: octylmaltoside; C_8_G: octylglucoside; β-CDx: β-cyclodextrin.

## Authors' contributions

Both authors were equally involved in the conceptualization and writing of this paper, and both have read and approved the final manuscript. JDO performed the literature search and PM developed the mathematical models.

## Supplementary Material

Additional file 1 **Studies of interactions of UCB with phospholipids**. Details of the three publications that were considered, including the degrees of supersaturation with UCB, the analytical methods used, the charateristics of the binding curve, the experimental problems, and the citation. Click here for file

Additional file 2 **Studies of interactions of UCB with alkyl saccharides and cyclodextrins**. Details of the three publications that were considered, including the degrees of supersaturation with UCB, the analytical methods used, the charateristics of the binding curve, the experimental problems, and the citation. Click here for file

Additional file 3 **Studies of interactions of UCB with bile salts**. Details of the seven publications that were considered, including the degrees of supersaturation with UCB, the analytical methods used, the charateristics of the binding curve, the experimental problems, and the citation. Click here for file

## References

[B1] Ostrow JD Mukerjee P Tiribelli C Structure and binding of unconjugated bilirubin: relevance for physiological and pathophysiological function J Lipid Res 1994 35 1715 1737 7852850

[B2] Mukerjee P Ostrow JD Review: Bilirubin pKa studies: new models and theories indicate high pKa values in water, dimethylformamide and DMSO BMC Biochemistry 2010 11 15 20350305 10.1186/1471-2091-11-15 PMC2880415

[B3] Boiadjiev SE Watters K Wolf S Lai BN Welch WH McDonagh AF Lightner DA pKa and aggregation of bilirubin: titrimetric and ultracentrifugation studies on water-soluble pegylated conjugates of bilirubin and fatty acids Biochemistry 2004 43 15617 15632 10.1021/bi0481491 15581375

[B4] Hahm JS Ostrow JD Mukerjee P Celic L Ionization and self-association of unconjugated bilirubin, determined by rapid solvent partition from chloroform, with further studies of bilirubin solubility J Lipid Res 1992 33 1123 1137 1431594

[B5] Ostrow JD Mukerjee P Revalidation and rationale for high pKa values of unconjugated bilirubin BMC Biochem 2007 8 7 10.1186/1471-2091-8-7 17475001 PMC1877803

[B6] Mukerjee P Ostrow JD Tiribelli C Low solubility of unconjugated bilirubin in dimethylsulfoxide - water systems: implications for pK_*a *_determinations BMC Biochemistry 2002 3 17 10.1186/1471-2091-3-17 12079498 PMC116679

[B7] Brodersen R Theilgaard J Bilirubin colloid formation in neutral aqueous solution Scand J Clin Lab Invest 1969 24 395 398 10.3109/00365516909080178 5375744

[B8] Wosiewitz U Schroebler S Solubilization of unconjugated bilirubin by bile salts Experientia 1979 35 717 718 10.1007/BF01968199 38133

[B9] Ostrow JD Celic L Mukerjee P Molecular and micellar associations in the pH-dependent stable and metastable dissolution of unconjugated bilirubin by bile salts J Lipid Res 1988 29 335 348 3379345

[B10] With TK Biology of Bile Pigments 1954 Copenhagen: Arne Frost-Hansen

[B11] With TK Bile Pigments: Chemical, Biological and Clinical Aspects 1968 New York: Academic Press

[B12] Tipping E Ketterer B Christodoulides L Binding to egg phosphatidylcholine of some organic anions (bromsulphophthalein, oestrone sulphate, haem and bilirubin) that bind to ligandin and aminoazo-dye binding protein A Biochem J 1979 180 327 337 39548 10.1042/bj1800327 PMC1161057

[B13] Kano K Ishimura T Properties of alkyl β-D-glucoside and alkyl β-D-maltoside micelles J Chem Soc Perkin Trans II 1995 1655 1660 10.1039/p29950001655

[B14] Kano K Arimoto S Ishimura T Conformational enantiomerism of bilirubin and pamoic acid induced by protonated aminocyclodextrins J Chem Soc Perkin Trans II 1995 1661 1667 10.1039/p29950001661

[B15] D'Alagni M Galantini L Giglio E Gavuzzo E Scaramuzza L Micellar aggregates of sodium glycocholate and sodium taurocholate and their interaction complexes with bilirubin-IXα J Chem Soc Faraday Trans 1994 90 1523 1532 10.1039/ft9949001523

[B16] Eriksen EF Danielsen H Brodersen R Bilirubin-liposome interaction. Binding of bilirubin dianion, protonization and aggregation of bilirubin acid J Biol Chem 1981 256 4269 4274 7194339

[B17] Lightner DA Holmes DL McDonagh AF Dissociation constants of water-insoluble carboxylic acids by ^13^C-NMR. *p *K_a_s of mesobiliverdin-XIIIα and mesobilirubin-XIIIα Experientia 1996 51 639 642 10.1007/BF01969747 8698102

[B18] Holmes DL Lightner DA Synthesis and acidity constants of ^13^CO_2_H-labelled dicarboxylic acids. *p*K_a_s from ^13^C-NMR Tetrahedron 1996 52 5319 5338 10.1016/0040-4020(96)00153-6

[B19] Lightner DA Holmes DL McDonagh AF On the acid dissociation constants of bilirubin and biliverdin. p*K*_a_values from ^13^C NMR spectroscopy J Biol Chem 1996 271 2397 2405 10.1074/jbc.271.5.2397 8576198

[B20] McDonagh AF Phimster A Boiadjiev SE Lightner DA Dissociation constants of carboxylic acids by ^13^C-NMR in DMSO/water Tetrahedron Letters 1999 40 8515 8518 10.1016/S0040-4039(99)01841-9

[B21] Hahm JS Mun GH Lee HL Eun CS Park JY Han DS Choi HS Ahn YH [Interactions of unconjugated bilirubin with bile acid by rapid solvent partition] Taehan Kan Hakhoe Chi 2002 8 80 89 12499820

[B22] Cestaro B Cervato G Ferrari S Di Silvestro G Monti D Manitto P Interaction of bilirubin with small unilamellar vesicles of dipalmitoylphosphatidylcholine Ital J Biochem 1983 32 318 329 6689320

[B23] Garvin JE Karnovsky ML The titration of some phosphatides and related compounds in a non-aqueous system J Biol Chem 1956 221 211 222 13345811

[B24] Jukes TH The electrometric titration of lecithin and cephalin J Biol Chem 1934 107 783 787

[B25] Carey MC Spivak W Ostrow JD Physical chemistry of bile pigments and porphyrins with particular reference to bile Bile Pigments and Jaundice; Molecular, Metabolic and Medical Aspects 1986 New York: Marcel Dekker 81 132

[B26] Zucker SD Gössling W Mechanism of hepatocellular uptake of albumin-bound bilirubin Biochim Biophys Acta 2000 1463 197 208 10.1016/S0005-2736(99)00196-0 10675499

[B27] Zucker SD Gössling W Bootle EJ Sterritt C Localization of bilirubin in phospholipid bilayers by parallax analysis of fluorescence quenching J Lipid Res 2001 42 1377 1388 11518756

[B28] Vazquez J Garcia-Calvo M Valdivieso F Mayer F Mayer FJ Interaction of bilirubin with the synaptosomal plasma membrane J Biol Chem 1988 263 1255 1265 3335545

[B29] Zucker SD Storch J Zeidel ML Gollan JL Mechanism of the spontaneous transfer of unconjugated bilirubin between small unilamellar phosphatidylcholine vesicles Biochemistry 1992 31 3184 3192 10.1021/bi00127a020 1554704

[B30] Vazquez J Ortega G Valdivieso F Mayor F Jr Interaction of bilirubin with gangliosides J Biochem (Tokyo) 1989 106 139 142 10.1093/oxfordjournals.jbchem.a122803 2777744

[B31] Amit Y Fedunec S Thomas PD Poznansky MJ Schiff D Bilirubin-neural cell interaction: characterization of initial cell surface binding leading to toxicity in the neuroblastoma cell line N-115 Biochim Biophys Acta 1990 1055 36 42 10.1016/0167-4889(90)90088-U 2223872

[B32] Rashid H Ali MK Tayyab S Effect of pH and temperature on the binding of bilirubin to human erythrocyte membranes J Biosci 2000 25 157 161 10878856

[B33] Sato H Kashiwamata S Interaction of bilirubin with human erythrocyte membranes Biochem J 1983 210 489 496 6860307 10.1042/bj2100489 PMC1154249

[B34] Bratlid D The effect of pH on bilirubin binding by human erythrocytes Scand J Clin Lab Invest 1972 29 453 459 10.3109/00365517209080265 20465481

[B35] Wennberg RP The importance of free bilirubin acid salt in bilirubin uptake by erythrocytes and mitochondria Pediatr Res 1988 23 443 447 10.1203/00006450-198804000-00021 3374999

[B36] Lightner DA Gawronski JK Gawronska K Conformational enantiomerism in bilirubin. Selection by cyclodextrins J Am Chem Soc 1985 107 2456 2461 10.1021/ja00294a042

[B37] Carey MC Bile acids and bile salts: ionization and solubility properties Hepatology 1984 4 66S 71S 10.1002/hep.1840040812 6479887

[B38] Kano K Tsujino N Kim M Mechanisms for steroid-induced conformational enantio-merism of bilirubin in protic solvents J Chem Soc Perkin Trans II 1992 1747 1752 10.1039/p29920001747

[B39] Harman AD Kibbey RG Sablik MA Fintschenko Y Kurtin WE Bushey MM Micellar electrokinetic capillary chromatography analysis of the behavior of bilirubin in micellar solutions J Chromatogr A 1993 652 525 533 10.1016/0021-9673(93)83274-V 8287141

[B40] Kurtin WE Enz J Dunsmoor C Evans N Lightner DA Acid dissociation constants of bilirubin and related carboxylic acid compounds in bile salt solutions Arch Biochem Biophys 2000 381 83 91 10.1006/abbi.2000.1949 11019823

[B41] D'Alagni M Delfini M Galantini L Giglio E A study of the interaction of bilirubin with sodium deoxycholate in aqueous solutions J Phys Chem 1992 96 10520 10528 10.1021/j100204a073

[B42] D'Alagni M D'Archivio AA Giglio E Scaramuzza L Structure of sodium and rubidium taurodeoxycholate micellar aggregates and their interaction complexes with bilirubin-IXα J Phys Chem 1994 98 343 353 10.1021/j100052a056

[B43] Brodersen R Prevention of kernicterus, based on recent progress in bilirubin chemistry Acta Paediatr Scand 1977 66 625 634 10.1111/j.1651-2227.1977.tb07959.x 19921

[B44] Brodersen R Binding of bilirubin to albumin; implications for prevention of bilirubin encephalopathy in the newborn CRC Crit Rev Clin Lab Sci 1979 11 305 399 6985857

[B45] Brodersen R Heirwegh KPM, Brown SB Physical chemistry of bilirubin: Binding to macromolecules and membranes Bilirubin. Chemistry 1982 1 Boca Raton, FL: CRC Press 75 123

[B46] Brodersen R Ostrow JD Aqueous solubility, albumin binding and tissue distribution of bilirubin Bile Pigments and Jaundice; Molecular, Metabolic and Medical Aspects 1986 New York: Marcel Dekker 157 181

[B47] Brodersen R Stern L Deposition of bilirubin acid in the central nervous system - A hypothesis for the development of kernicterus Acta Paediatr Scand 1990 79 12 19 10.1111/j.1651-2227.1990.tb11323.x 2180252

[B48] Ostrow JD Pascolo L Shapiro SM Tiribelli C New concepts in bilirubin encephalopathy Eur J Clin Invest 2003 33 988 997 10.1046/j.1365-2362.2003.01261.x 14636303

[B49] Ostrow JD Pascolo L Brites D Tiribelli C Molecular basis of bilirubin-induced neurotoxicity Trends Mol Med 2004 10 65 70 10.1016/j.molmed.2003.12.003 15102359

