# Supplementary material for: Interactions of unconjugated bilirubin with vesicles, cyclodextrins and micelles: New modeling and the role of high pKa values
Source: BMC Biochem. 2010 Mar 29;11:16. doi: 10.1186/1471-2091-11-16 (PMC2868783; doi:10.1186/1471-2091-11-16)
Supplement: Additional file 1 — Studies of interactions of UCB with phospholipids. Details of the three publications that were considered, including the degrees of supersaturation with UCB, the analytical methods used, the charateristics of the binding curve, the experimental problems, and the citation. [file 1471-2091-11-16-S1.DOC]

##

**Table S1. Studies of interactions of UCB with phospholipids**

| **Binder** | **Aq. Saturation ratios (R) for bilirubin at pH’s*** | **Method** | **Characteristics of Binding Curve** | **Experimental**  **Problems**† | **Citation** |
| --- | --- | --- | --- | --- | --- |
| Phosphatidyl-choline vesicles | PL 100 *µ*M, UCB 10 *µ*M. R=101 at pH 6.5, 7 at pH 8.0. | Spectrophotometric  titration | Midpoint pH ~7.8 | A,B,C | Tipping, 1979 [1] |
| Phospholipid vesicles | Progressive UCB aggregation reported at pH < 8.2 | Fluorescence quenching, light-scattering, gradient centrifugation | As pH 🢁, binding  🢁 early and 🢃 late | B,C,D,E,F,G | Eriksen, 1981[2] |
| Gangliosides | Initial [UCB] = 2 - 20 *µ*M  (all supersaturated).  Gangliosides carry  negative charges | Differential absorption spectra | Binding 🢁 as pH 🢁 from 7.0 to 7.4 | A,B,D,F,H | Vazquez, 1989 [3] |

*Footnotes:* *****Calculated as ratio to saturation concentration at given pH, based on data from Hahm *et al.,* 1992 [4].

† **Experimental Problems** as listed below:

A - UCB not purified and/or its purity not documented

B - UCB not protected against degradation by exclusion of oxygen and light, by removal of oxidants from solvents,

and/or by avoidance of prolonged standing in strongly alkaline stock solutions.

C - Binder and/or solvents not pure.

D - Equilibrium not achieved and/or not attained rapidly.

E - Used prolonged analytical procedures.

F - UCB concentrations above saturation, with aggregation or precipitation and/or soluble multimers not accounted for.

G - Fluorescent probes added to vesicles, with unknown effects on UCB binding.

H - pH range examined not sufficiently wide, and/or insufficient numbers of data points.

**References**

1. Tipping E, Ketterer B, Christodoulides L: **Binding to egg phosphatidylcholine of some organic anions (bromsulphophthalein, oestrone sulphate, haem and bilirubin) that bind to ligandin and aminoazo-dye binding protein A**. *Biochem J* 1979, **180**: 327-337.

2. Eriksen EF, Danielsen H, Brodersen R: **Bilirubin-liposome interaction. Binding of bilirubin dianion, protonization and aggregation of bilirubin acid**. *J Biol Chem* 1981, **256**: 4269-4274.

3. Vazquez J, Ortega G, Valdivieso F, Mayor F, Jr.: **Interaction of bilirubin with gangliosides**. *J Biochem (Tokyo)* 1989, **106**: 139-142.

4. Hahm JS, Ostrow JD, Mukerjee P, Celic L: **Ionization and self-association of unconjugated bilirubin, determined by rapid solvent partition from chloroform, with further studies of bilirubin solubility**. *J Lipid Res* 1992, **33**: 1123-1137.
