# Supplementary material for: Interactions of unconjugated bilirubin with vesicles, cyclodextrins and micelles: New modeling and the role of high pKa values
Source: BMC Biochem. 2010 Mar 29;11:16. doi: 10.1186/1471-2091-11-16 (PMC2868783; doi:10.1186/1471-2091-11-16)
Supplement: Additional file 2 — Studies of interactions of UCB with alkyl saccharides and cyclodextrins. Details of the three publications that were considered, including the degrees of supersaturation with UCB, the analytical methods used, the charateristics of the binding curve, the experimental problems, and the citation. [file 1471-2091-11-16-S2.DOC]

##

**Table S2. Studies of interactions of UCB with alkyl saccharides and cyclodextrins.**

| **Binder** | **Aq. Saturation ratios (R) for bilirubin at pH’s*** | **Method** | **Characteristics of Binding Curve** | **Experimental**  **Problems**‡ | **Citation** |
| --- | --- | --- | --- | --- | --- |
| Dodecylmaltoside (C_12_M) micelles | Probably supersaturated; UCB 20 *µ*M, C_12_M 1 mM; Ka not given | CD spectra vs. pH (4-10) | Midpoint pH ~7.5 | F | Kano, 1995 [5] |
| β-cyclodextrin &  1-Amino- β-cyclodextrin | β-CDx R>400 at pH 7.0, R>200 at pH 8.0.  Amino-β-CDx probably supersaturated at all pHs. | CD spectra vs. pH (5.5-10.8) | Midpoint pH:  ~7.9 (β-CDx);  ~7.3 (Amino-β-CDx)† | F | Kano, 1995 [6] |
| α, β & γ-cyclodextrins  (α, β & γ-CDx) | Supersaturation higher than in Kano’s study of  β-CDx, since [UCB] higher and [β-CDx] the same. | CD spectra vs. pH at 10.0, 9.0, 8.0, 7.0 (6.0). | For β & γ-CDx), big 🡻 in CD with pH 🡻 from 8.0🡺 7.0. For β-CDx, no CD at pH 6.0. For α-CDx, no CD at any pH value. | C,F,H | Lightner, 1985 [7] |

*Abbreviations*: CD, circular dichorism (ellipticity)

*Footnotes:* *****Calculated as ratio to saturation concentration at given pH, based on data from Hahm *et al.,* 1992 [4].

† 1-amino, β-cyclodextrin precipitated at pH > 8.0.

‡ - **Experimental Problems:** C, F and H defined in footnotes to Supplementary Table 1.

References

4. Hahm JS, Ostrow JD, Mukerjee P, Celic L: **Ionization and self-association of unconjugated bilirubin, determined by rapid solvent partition from chloroform, with further studies of bilirubin solubility**. *J Lipid Res* 1992, **33**: 1123-1137.

5. Kano K, Ishimura T: **Properties of alkyl b-D-glucoside and alkyl b-D-maltoside micelles**. *J Chem Soc Perkin Trans II* 1995, 1655-1660.

6. Kano K, Arimoto S, Ishimura T: **Conformational enantiomerism of bilirubin and pamoic acid induced by protonated aminocyclodextrins**. *J Chem Soc Perkin Trans II* 1995, 1661-1667.

7. Lightner DA, Gawronski JK, Gawronska K: **Conformational enantiomerism in bilirubin. Selection by cyclodextrins**. *J Am Chem Soc* 1985, **107**: 2456-2461.
