# Supplementary material for: Interactions of unconjugated bilirubin with vesicles, cyclodextrins and micelles: New modeling and the role of high pKa values
Source: BMC Biochem. 2010 Mar 29;11:16. doi: 10.1186/1471-2091-11-16 (PMC2868783; doi:10.1186/1471-2091-11-16)
Supplement: Additional file 3 — Studies of interactions of UCB with bile salts. Details of the seven publications that were considered, including the degrees of supersaturation with UCB, the analytical methods used, the charateristics of the binding curve, the experimental problems, and the citation. [file 1471-2091-11-16-S3.DOC]

## **Supplementary Table 3 - Studies of interactions of UCB** with bile salts

| **Binder** | **Aq. Saturation ratios (R) for bilirubin at pH’s*** | **Method** | **Characteristics of Binding Curve** | **Experimental**  **Problems**† | **Citations** |
| --- | --- | --- | --- | --- | --- |
| Na Taurocholate, (TC) 50 mM | Always undersaturated. | Solvent partition vs. pH, CHCl_3_ vs. aq. buffered TC. | pKa's = 6.7 ± 0.5 and 7.2 ± 0.5 for TC-bound UCB | All criteria met | Hahm, 2002 [8]; Ostrow, 1994 [9] |
| Cholate, TC & TDC, 25 mM in PO_4_-BO_3_ buffers | Unknown – Final [UCB] not stated, but sometimes precipitates formed | Micellar electrokinetic capillary chromatograpy | pKa's = 6.2 & 6.6 | B,C,I Assumptions limit validity | Harman, 1993 [10] |
| TDC or TC  40 mM in DMSO/water (20 or 10% v/v) | Unknown – Final [MBR] not stated. | NMR titration of ^13^C-COOH labeled mesobilirubin-XIIIα. | DMSO pK'a 20%v/v 6.3, 6.7 10%v/v 5.9, 6.5 | B,C,E,I,J,K,L | Kurtin 2000 [11] |
| NaTDC 50 mM | 4.6 *µ*M UCB, R˜1 at pH 7.5.  34 *µ*M UCB, R>1 at pH < 8.3 | Circular Dichroism | CD spectra invert at pH ˜8.5 | A,F (at 34 *µ*M UCB),H | D’Alagni, 1992 [12] |
| Na Deoxycholate  50 mM | 4.6 *µ*M UCB, R˜1 at pH 7.5 | Circular Dichroism | CD spectra invert at pH > 8.8 | A,H | D’Alagni, 1994 [13] |
| NaTC & NaGC 50 mM | 4.6 *µ*M UCB, R˜1 at pH 7.5.  34 *µ*M UCB, R>1 at pH < 8.3 | Circular Dichroism | CD spectra invert at pH > 7.8-8.0 | A,F,H | D’Alagni, 1994 [14] |

*Abbreviations*: DMSO, dimethyl sulfoxide; MBR, mesobilirubin; TC, taurocholate; TDC, taurodeoxycholate; GC, glycocholate.

*Footnotes:* *****Calculated as ratio to saturation concentration at given pH, based on data from Hahm *et al.,* 1992 [4].

† **Experimental Problems:** A,B,C,E,F and H defined in footnotes to Supplementary Table 1.

I - No direct measurements of UCB pKa in aqueous systems. J - Incorrect pKa values used for reference carboxylic acids.

K - pH measurements not standardized in mixed solvents

L - Inappropriate extrapolation from mixed aqueous-organic solvent to pure water

**References**

4. Hahm JS, Ostrow JD, Mukerjee P, Celic L: **Ionization and self-association of unconjugated bilirubin, determined by rapid solvent partition from chloroform, with further studies of bilirubin solubility**. *J Lipid Res* 1992, **33**: 1123-1137.

8. Hahm JS, Mun GH, Lee HL, Eun CS, Park JY, Han DS *et al*.: **[Interactions of unconjugated bilirubin with bile acid by rapid solvent partition]**. *Taehan Kan Hakhoe Chi* 2002, **8**: 80-89.

9. Ostrow JD, Mukerjee P, Tiribelli C: **Structure and binding of unconjugated bilirubin: relevance for physiological and pathophysiological function**. *J Lipid Res* 1994, **35**: 1715-1737.

10. Harman AD, Kibbey RG, Sablik MA, Fintschenko Y, Kurtin WE, Bushey MM: **Micellar electrokinetic capillary chromatography analysis of the behavior of bilirubin in micellar solutions**. *J Chromatogr A* 1993, **652**: 525-533.

11. Kurtin WE, Enz J, Dunsmoor C, Evans N, Lightner DA: **Acid dissociation constants of bilirubin and related carboxylic acid compounds in bile salt solutions**. *Arch Biochem Biophys* 2000, **381**: 83-91.

12. D'Alagni M, Delfini M, Galantini L, Giglio E: **A study of the interaction of bilirubin with sodium deoxycholate in aqueous solutions**. *J Phys Chem* 1992, **96**: 10520-10528.

13. D'Alagni M, D'Archivio AA, Giglio E, Scaramuzza L: **Structure of sodium and rubidium taurodeoxycholate micellar aggregates and their interaction complexes with bilirubin-IXa**. *J Phys Chem* 1994, **98**: 343-353.

14. D'Alagni M, Galantini L, Giglio E, Gavuzzo E, Scaramuzza L: **Micellar aggregates of sodium glycocholate and sodium taurocholate and their interaction complexes with bilirubin-IXa**. *J Chem Soc Faraday Trans* 1994, **90**: 1523-1532.
